# *Btbd8* deficiency reduces susceptibility to colitis by enhancing intestinal barrier function and suppressing inflammation

**DOI:** 10.3389/fimmu.2024.1382661

**Published:** 2024-03-15

**Authors:** Xiaoqiong Yang, Zichan He, Qiman Dong, Shanshan Nai, Xiaowei Duan, Jiayu Yu, Nannan Zhao, Xiaoling Du, Lingyi Chen

**Affiliations:** Institute of Translational Medicine, Tianjin Union Medical Center, State Key Laboratory of Medicinal Chemical Biology, Tianjin Key Laboratory of Protein Sciences, Frontiers Science Center for Cell Responses, National Demonstration Center for Experimental Biology Education and College of Life Sciences, Nankai University, Tianjin, China

**Keywords:** *BTBD8*, intestinal barrier integrity, DSS, inflammation, inflammatory bowel disease (IBD)

## Abstract

**Introduction:**

*BTBD8* has been identified as a susceptible gene for inflammatory bowel diseases (IBD). However, the function of *BTBD8* in normal development and IBD pathogenesis remains unknown.

**Methods:**

We administered drinking water with 3% dextran sodium sulfate (DSS) to wild-type (WT) and *Btbd8* knockout (KO) mice for seven consecutive days to induce IBD. Subsequently, we further examined whether *Btbd8* KO affects intestinal barrier and inflammation.

**Results:**

We demonstrated that *Btbd8* deficiency partially protects mice from DSS-induced IBD, even though no obvious phenotypes were observed in *Btbd8* KO mice. *Btbd8* deletion leads to strengthened tight junctions between intestinal epithelial cells, elevated intestinal stem cell activity, and enhanced mucus layer. All these three mechanisms work together to improve the intestinal barrier integrity in *Btbd8* KO mice. In addition, *Btbd8* deficiency mitigates inflammation by reducing the expression of IL-1β and IL-6 by macrophages.

**Discussion:**

Our studies validate the crucial role of *Btbd8* in IBD pathogenesis, and reveal that *Btbd8* deficiency may ameliorate DSS-induced IBD through improving the intestinal barrier integrity, as well as suppressing inflammatory response mediated by macrophages. These findings suggest that *Btbd8* could be a promising therapeutic target for the treatment of IBD.

## Introduction

Inflammatory bowel disease (IBD), comprising Crohn’s disease (CD) and ulcerative colitis (UC), is an idiopathic chronic intestinal disease, characterized by impaired intestinal barrier and chronic relapsing intestinal inflammation (1, 2). Recent studies have suggested that various factors, including genetic factors, immune response, microbiota, and environment, contribute to the pathogenesis of IBD (3–6).

Through genome-wide association studies (GWAS), more than 200 IBD susceptibility genes and loci have been identified (7–10). Analysis of these IBD susceptibility genes and loci revealed several pathways crucial for IBD predisposition, including barrier function, epithelium reconstitution, microbial defense, and regulation of immunity. For example, *C1orf106* and *RNF186* are involved in maintaining the barrier integrity (11, 12), *HNF4A*, *NKX2*-3, and *STAT3* contribute to the regeneration of intestinal epithelium (13–16), *CARD9* and *NOD2* play roles in microbe-sensing (17–19), and *IL10* and *IL23R* regulate immune response (20, 21). These genetic data suggest that the interaction between the intestinal microbiome and the intestinal immune system plays a key role in IBD pathogenesis.

Despite extensive studies on IBD risk genes, the functions and mechanisms of some IBD susceptibility genes, including *BTBD8* (BTB domain containing 8), remain elusive. *BTBD8* was identified as an IBD susceptibility gene by a trans-ancestry GWAS analysis. Single-cell RNA sequencing revealed that *BTBD8* expression is decreased in UC patients, particularly in intestinal epithelial cells (IECs) (22), implying a potential role of *BTBD8* in IBD pathogenesis. In addition, it has been shown that Btbd8, also known as APache, is associated with clathrin-coated vesicles, and regulates synaptic vesicle trafficking, neuronal development, and synaptic plasticity (23). Nevertheless, whether and how BTBD8 regulates normal development and IBD pathogenesis remains unexplored.

To investigate the potential role of Btbd8 in normal development and IBD pathogenesis, we generated *Btbd8* knockout (KO) mice with CRISPR/Cas9. *Btbd8* KO mice are healthy and fertile, and exhibit reduced susceptibility to dextran sodium sulfate (DSS)-induced IBD. Mechanistically, *Btbd8* KO suppresses endocytosis in epithelial cells, hence elevating the expression of tight junction proteins ZO-1 and Occludin. *Btbd8* KO also increases the number of intestinal stem cells (ISCs) and goblet cells, promoting epithelial regeneration and mucus formation respectively. Therefore, the intestinal barrier function is enhanced in *Btbd8* KO mice. In addition, *Btbd8* KO reduces the expression of *IL-1β* and *IL-6* in macrophages, thus suppressing inflammation. Collectively, our data reveal that Btbd8 facilitates IBD pathogenesis, and might be a therapeutic target for IBD treatment.

## Materials and methods

### Mice and administration

*Btbd8* KO mice were constructed in Nankai animal resources center. Cas9 protein and two guide RNAs targeting sites flanking the exon 14 of *Btbd8*, were microinjected into zygotes. The surviving embryos were transferred into pseudo-pregnant female mice. To identify KO mice, the mouse genotype was determined via PCR analysis using two pairs of specific primers and genomic DNA extracted from the mouse tail. WT mice were purchased from Vital River Laboratory Animal Technology Co., Ltd. (Beijing, China). All mice were in a C57BL/6 background. Unless otherwise specified, experiments were conducted using female mice aged 6-10 weeks. Age- and sex-matched littermates were used as control mice. All animal experiments were carried out with a strict accordance with institutional guideline, and the use of mice for this research is approved by Nankai Animal Care and Use Committee (Approval number: 2021-SYDWLL-1-0001).

### DSS-induced colitis

The age- and sex-matched WT and *Btbd8* KO mice were provided with drinking water containing 3% DSS (MP Biomedicals, 160110) for 7 days. Body weight of mice was monitored daily, and changes were calculated relative to the initial body weight. Every mouse underwent daily assessment using the DAI score, which includes weight loss, stool consistency, and the presence of fecal blood. The scoring system for calculating DAI was described previously (24). Body weight loss was assessed using the following scale: 0 for none, 1 for 1–5%, 2 for 6–10%, 3 for 11–18%, and 4 for >18%. Stool consistency was assessed using the following scale: 0 for normal, 1 for soft but still formed stools, 2 for soft stools, 3 for very soft or wet stools, and 4 for watery diarrhea stools. Stool bleeding was assessed using the following scale: 0 for normal, 1 for a brown color, 2 for occult blood, 3 for visible blood trace, and 4 for gross bleeding.

### Histology and histopathological score

For each mouse, the entire colon was promptly excised, and its length was measured. The distal section of colon was fixed in 4% paraformaldehyde for 24 h and embedded in paraffin or Tissue-Tek OCT compound. Other sections were collected for qRT-PCR or Western blot. Five-micron paraffin sections were deparaffinized and stained with hematoxylin and eosin (H&E) for histological analysis and assessment of the histopathological score. HE staining followed the standard processing procedures and the scoring system for calculating histopathological score was described previously (25). Colon sections were assessed using the following scale: 0 for normal tissue; 1 for mild inflammation in the mucosa with some infiltrating mononuclear cells; 2 for increased level of inflammation in the mucosa with more infiltrating cells, damaged crypt glands and epithelium, mucin depletion from goblet cells; 3 for extensive infiltrating cells in the mucosa and submucosa area, crypt abscesses present within creased mucin depletion, and epithelial cell disruption; 4 for massive infiltrating cells in the tissue, complete loss of crypt.

### Immunofluorescence

Paraffin sections were deparaffinized, and antigen retrieval was achieved by boiling in citrate antigen retrieval solution before analysis. Frozen sections were equilibrated at room temperature for 30 min, and then immersed in PBS for 15 min to remove OCT before analysis. Both paraffin and frozen sections were incubated in 5% goat serum for 45 min at room temperature. Subsequently, primary antibodies treatment was conducted in PBS supplemented with 0.3% Triton X-100 overnight at 4°C.

For immunofluorescence with transwell inserts, inserts (Corning) were incubated in methyl alcohol at -20°C overnight. Subsequently, the inserts were transferred into acetone (-20°C) for 1 min, and then incubated in blocking buffer (containing PBS, 2% goat serum, 1% BSA, 0.1% cold fish skin gelatin, 0.1% Triton X-100, and 0.05% Tween-20) for 1 hour. Next, the inserts were incubated with specific primary antibodies in PBS, supplemented with 1% BSA, and 0.1% cold fish skin gelatin at 4°C overnight.

Primary antibodies were normal Rabbit IgG (Cell Signaling Technology, 2729), anti-ZO-1 (Proteintech, 21733), anti-Occludin (Proteintech, 27260), anti-Muc2 (Proteintech, 27675), normal Mouse IgG (Cell Signaling Technology, 5415) and anti-Lgr5 (Sigma, MA5-25644). Primary antibodies were visualized using fluorophore-conjugated secondary antibodies, FITC-goat anti-Rabbit IgG (Invitrogen, A11034) or FITC-goat anti-Mouse IgG (Invitrogen, A11029), for 45 min at room temperature. DAPI was used for nuclear staining. Microscopic images were captured using either upright fluorescence microscope Axio Imager Z1 (Zeiss) or Laser scanning confocal microscope LSM710 (Zeiss).

### Immunohistochemical staining

Paraffin sections were deparaffinized and subjected to antigen retrieval by boiling in a citrate antigen retrieval solution. The sections were incubated at room temperature in 5% goat serum for 45 minutes, and subsequently incubated with anti-Ki-67 antibody (Proteintech, 27309) in PBS containing 0.3% Triton X-100 at 4°C overnight. A 3% hydrogen peroxide treatment for 10 min was applied to remove endogenous peroxidases. The sections were then incubated with goat anti-rabbit IgG conjugated with horseradish peroxidase (HRP) (ORIGENE, ZB-2301) for 45 min at room temperature. The HRP activity was visualized using a diaminobenzidine solution (ORIGENE, ZLI-9018), and the sections were counterstained with hematoxylin. Microscopic images were captured using the Leica DM3000 microscope (Leica).

### Transmission electron microscope (TEM)

The distal sections of colon were fixed overnight at 4°C with 2.5% glutaraldehyde. The subsequent standard processing procedures, including fixation in osmium acid, dehydration, embedding, sectioning, and co-staining with yellow acetate and lead citrate, were carried out by Shiyanjia Lab (Hangzhou, China). The ultrastructure was observed using a transmission electron microscope H-7650 (Hitachi).

### Quantitative reverse-transcription PCR (qRT-PCR)

Total RNA was extracted from cells or tissues using a RNeasy Mini Kit (Qiagen) or TRIZOL (Invitrogen), and converted to cDNA using a Reverse Transcription kit (GenStar) according to the manufacturer’s instruction. The PCR reactions were performed with a RealStar Probe Fast Mixture kit (GenStar) on a Quantitative PCR machine (Bio-Rad). The primers are listed in **Supplementary Table S1**.

### Western blot

Tissues were harvested and resuspended in RIPA lysis buffer (20 mM Tris-HCl pH 8.0, 60 mM NaCl, 0.2% glycerol, 0.02% NP-40, 0.04 mM EDTA) supplemented with protease inhibitors. The suspension was then sonicated for 30 seconds and subsequently centrifuged at 12,000 rpm for 15 min at 4°C. The protein concentration was measured using a BCA Protein Assay Kit (Solarbio). The total protein samples were separated on SDS-PAGE Gel and transferred to a PVDF membrane (Cytiva). Membranes were blocked for 2 hours at room temperature with blocking buffer and incubated overnight with specific primary antibodies at 4°C. The primary antibodies were anti-pro-IL-1β (Abclonal, A11370), anti-IL-6 (Abclonal, A0286), and anti-β-Tubulin (Abmart, M20005H). Bound primary antibodies were detected by HRP-conjugated secondary antibodies, donkey anti-Rabbit IgG (Cytiva, NA934V) and sheep anti-Mouse IgG (Cytiva, NA931V). Images were captured using an automated chemiluminescence imaging analysis system (Tanon).

### Intestinal permeability assay

After an 8-hour fasting period, 0.6 mg/g bodyweight of FITC-dextran (Sigma, 46944) was administered to mice via gavage. Four hours later, whole blood was collected and subsequently centrifuged twice at 12,000 rpm for 3 min at 4°C to obtain serum. The serum was then transferred into 96-well microplates (Corning), and FITC levels were quantified using fluorometry at 490 nm with a microplate reader Enspire (PerkinElmer). FITC-dextran was diluted with PBS to create a standard curve for calculating the FITC concentration.

### Isolation of intestinal epithelial cells

The entire colon was dissected out and longitudinally incised. The intestinal lumen was cleansed of fecal content using ice-cold HBSS. Subsequently, the colon was cut into 1 cm-long segments, which were then transferred into a 50 ml centrifuge tube and incubated in isolation buffer (consisting of HBSS, 5 mM EDTA, 1 mM DTT) for 15 min at 37°C on a shaker. The tube was then placed on ice for 15 min, followed by thorough vortexing for 1 min. The suspension was carefully collected. To release more epithelial cells, the pellet was washed with ice-cold HBSS and vigorously shaken. The suspension was then collected, and this process was repeated twice. Epithelial cells were harvested by centrifugation at 1200 g for 5 min at 4°C.

### Organoids isolation and culture

Crypt isolation and organoid culture were performed as described previously (26). The entire small intestine was removed from the mouse abdominal cavity, and the contents of intestinal lumen were flushed with ice-cold PBS. The villi were removed from the surface of the small intestine by scraping with a sterile glass slide. Then crypts were released from the small intestine by incubating with PBS containing 2 mM EDTA for 30 min at 4°C while rocking on a shaker. Then crypts were harvested by centrifugation at 290 g for 5 min at 4°C. 50 μl of Matrigel (Corning)/crypt suspension, containing 400-500 crypts, was added to the center of each 37°C pre-warmed 24-well plate. The plate was transferred to a 37°C incubator for 20 min to allow the Matrigel dome to solidify. Then 450 μl IntestiCult™ mouse organoid growth medium (STEMCELL Technologies) was added to each well. The plate was placed in a 37°C incubator with 5% CO_2_.

### Generation of intestinal organoid-derived monolayers

Mouse organoids were transitioned to IntestiCult™ human organoid growth medium (hOGM, STEMCELL Technologies) at least one generation before being converted to monolayer culture. 24-well transwell filters were coated with 5% Matrigel at 37°C for 2-4 hours. Two to three well-formed organoid domes were flushed and resuspended using Gentle Cell Dissociation Reagent (STEMCELL Technologies), followed by centrifugation at 200 g for 3 min at 4°C. The organoids were then incubated in 0.05% Trypsin-EDTA for 15 min at 37°C and mechanically disrupted by pipetting to obtain a single-cell or small fragments suspension. Subsequently, 1 ml of DMEM/F12 (Gibco) was added to the suspension, which was then centrifuged at 290 g for 5 min at 4°C. After removing supernatant, cells were resuspended in 100 μl of hOGM with 10 μM Y-27632 (Selleck Chemicals) and were seeded on the transwell insert. Additionally, 600 μl of hOGM with 10 μM Y-27632 were gently added into the lower compartment. The medium was refreshed every 2–3 days, and monolayers were maintained for up to 7 days.

### Isolation and culture of BMDMs

The isolation and differentiation of BMDMs were performed as previously described (27). Mouse bone marrow was obtained from the femur and tibia bones, followed by centrifugation at 1500 rpm for 5 min to collect the BM cells. Red blood cells were lysed using 5 ml of ACK buffer (Solarbio). The cells were then plated onto two 24-well plates in 0.5 ml of BMDM medium (consisting of DMEM, 20% FBS, 1% P/S, and 20 ng/ml M-CSF) and cultured for 7 days, resulting in a confluent layer of macrophages. Subsequently, the BMDMs were stimulated with 100 ng/ml LPS (Sigma) and 20 g/ml IFN-γ (R&D Systems) to induce M1 polarization.

### Statistical analysis

Data were analyzed using GraphPad Prism software and were presented as means ± SEM. Statistical analysis was performed with unpaired two-tailed Student’s *t* test. Statistically significant p-values were represented in figures as follows: *, p < 0.05; **, p < 0.01; ***, p < 0.001.

## Results

### *Btbd8* deficiency reduces susceptibility to DSS-induced IBD

**Figure 1:**
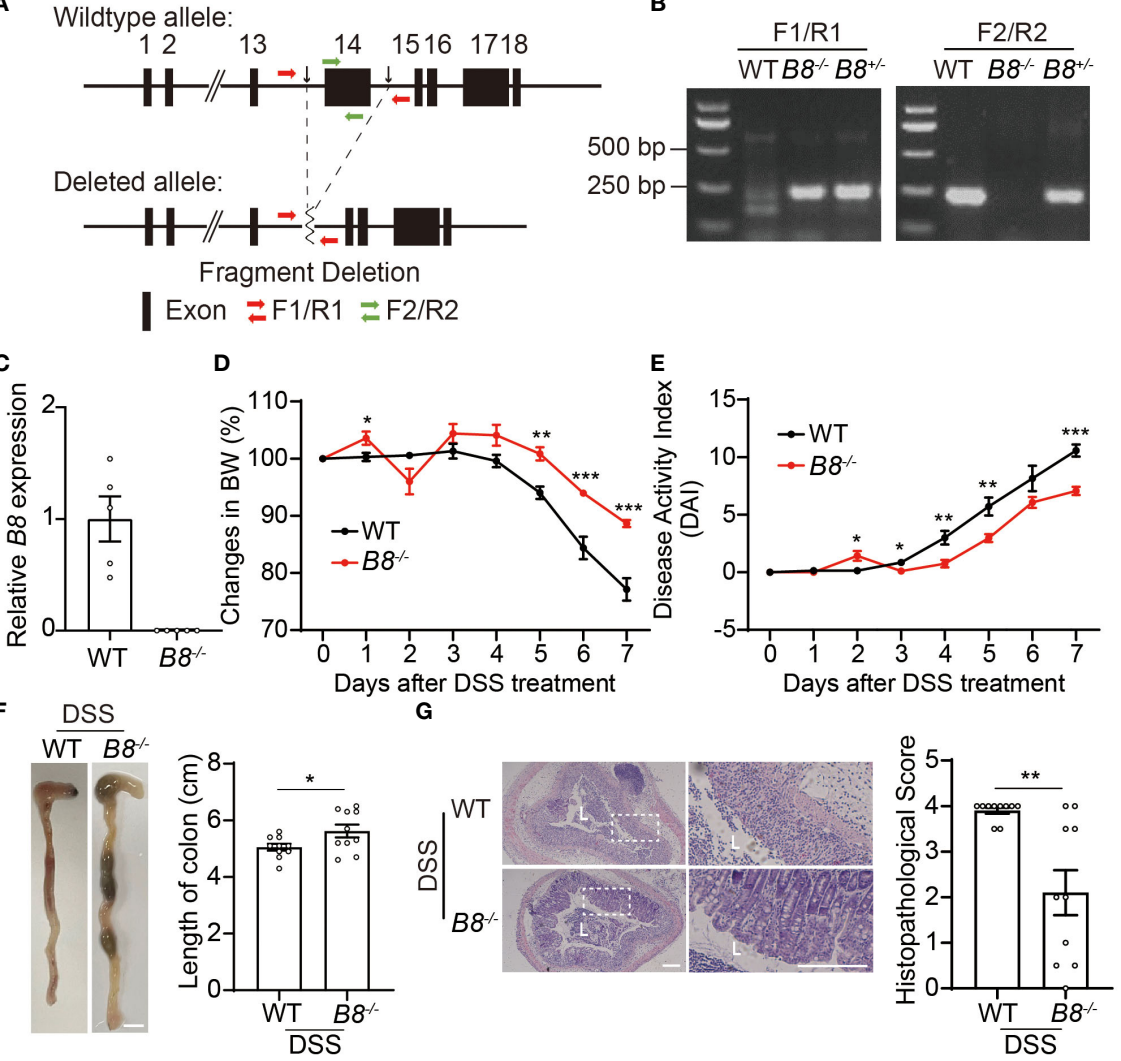
*Btbd8* KO mice are partially resistant to DSS-induced IBD. **(A)** Schematic illustration of the construction of *Btbd8* KO mice. Two black arrows mark the sgRNA targeting sites. **(B)** Genotyping of WT, *Btbd8* ^+/-^, and *Btbd8* ^-/-^ mice using F1/R1 and F2/R2 primers indicated in **(A)**. **(C)** qRT-PCR validates the knockout of *Btbd8* (n=5). **(D, E)** Body weight changes **(D)** and DAI scores **(E)** in WT and *Btbd8* KO mice with DSS treatment (n=7). **(F)** Representative images of colon (left panel) and the length of colon (right panel) in WT and *Btbd8* KO mice after 7-day DSS treatment (n=10). Scale bar: 0.5 cm. **(G)** Representative histopathological images of colon tissue sections (left panel) and the histopathological score (right panel) in WT and *Btbd8* KO mice after 7-day DSS treatment (n = 10). The lumen of intestine is indicated by L. Magnified images are shown in the right side. Scale bar: 200 μm. Data were presented as means ± SEM. *, p < 0.05; **, p < 0.01; ***, p < 0.001.

To investigate the function of Btbd8 in development and IBD, 1 homozygous and 6 heterozygous *Btbd8* knockout (KO) mice were generated by zygotic microinjection of Cas9 and two sgRNAs targeting two sites flanking *Btbd8* exon 14 (**Figures 1A–C**, **Supplementary Figures S1A, B**). The homozygous *Btbd8* KO mouse was used as the founder mouse for the subsequent mating and study. Mating between male and female *Btbd8* ^+/-^ mice yields *Btbd8* ^+/+^, *Btbd8* ^+/-^, and *Btbd8* ^-/-^ progenies, close to the expected Mendelian ratio of 1:2:1 (**Supplementary Figure S1C**). No morphological and histopathological abnormalities were noticed in various organs, including intestine, lung, heart, liver, spleen, and kidney (**Supplementary Figure S1D**).

Next, we attempted to address whether *Btbd8* deficiency affects the development of colitis. To induce IBD, we administered 3% DSS in drinking water to both wild-type (WT) and *Btbd8* KO mice for 7 days, and monitored colitis symptoms in these mice. We found that compared with DSS treated WT mice, DSS treated *Btbd8* KO mice exhibit milder colitis symptoms, such as less body weight loss, decreased Disease Activity Index (DAI) score, and longer colon (**Figures 1D–F**), whereas no difference in body weight and colonic length between untreated WT and *Btbd8* KO mice was observed (**Supplementary Figures S1E, F**). Furthermore, histological analysis revealed that more severe histological damage occurs in the colon of WT mice than in *Btbd8* KO mice (**Figure 1G**). In summary, our results suggest that even though *Btbd8* is dispensable for normal development, it is involved in the development of IBD.

### Enhanced barrier integrity in *Btbd8* KO mice upon DSS treatment

**Figure 2:**
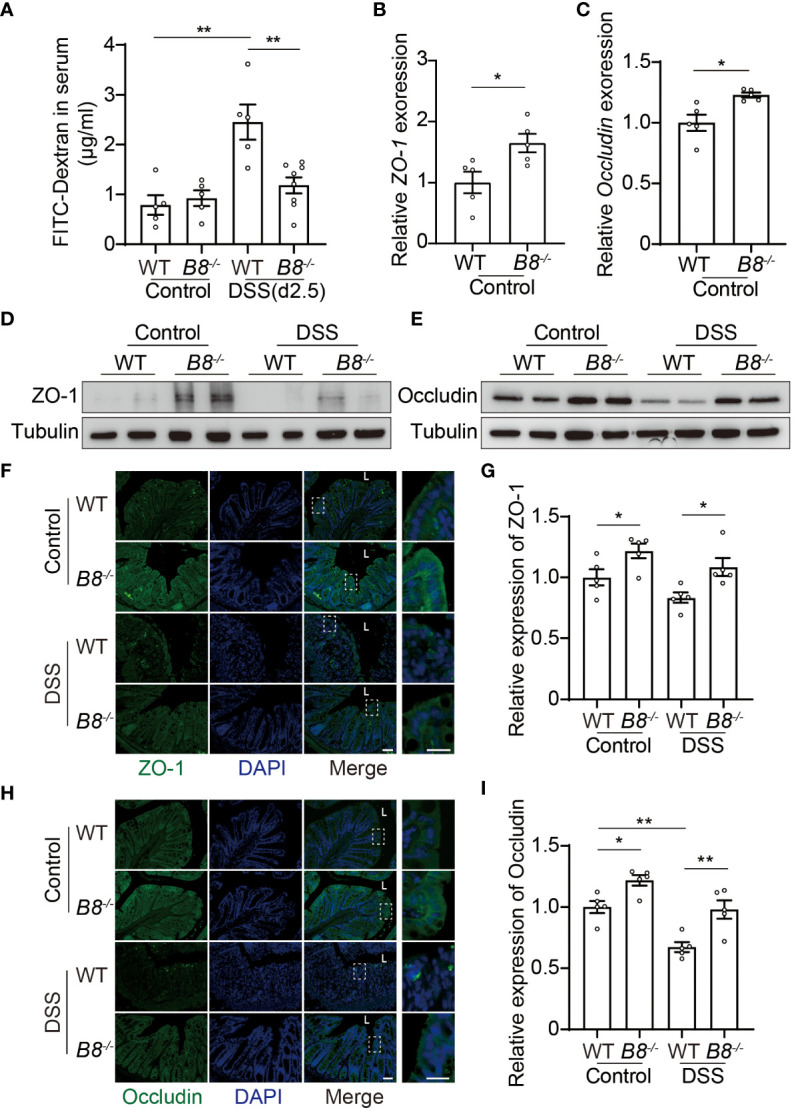
*Btbd8* deficiency reduces intestinal permeability, and enhances the expression of tight junctional proteins, ZO-1 and Occludin. **(A)** Intestinal permeability assay was performed in WT and *Btbd8* KO mice on day 0 (Control) and day 2.5 of DSS treatment (n=5-8). **(B, C)** The expression of *ZO-1* **(B)** and *Occludin* **(C)** in isolated WT and *Btbd8* KO IECs was analyzed by qRT-PCR (n=5). **(D, E)** Expression of ZO-1 and Occludin proteins in isolated WT and *Btbd8* KO IECs were detected by Western blot. **(F)** to **(I)** Immunofluorescence staining of colonic sections from untreated mice and mice with 7-day DSS treatment. **(F, H)** Representative images of ZO-1 **(F)** and Occludin **(H)** staining. The lumen of intestine is indicated by L. Magnified images are shown in the right side. Scale bar: 50 μm. **(G, I)** Quantification of ZO-1 **(G)** and Occludin **(I)** immunofluorescence (n=5). Data were presented as means ± SEM. *, p < 0.05; **, p < 0.01.

The intestinal epithelium separates gut microbiota from the host immune system, and is vital for preventing immune response to non-pathogenic commensals and dietary antigens (28, 29). Given that the expression level of *BTBD8* in intestinal epithelium is higher than those in immune cells and stroma cells (**Supplementary Figure S2**), we hypothesized that KO of *Btbd8* might improve the integrity of the intestinal barrier, leading to reduced susceptibility to IBD. DSS induced colitis symptoms are more severe in WT mice than in *Btbd8* KO mice (**Figures 1D–G**), which might result in more impaired intestinal barrier in DSS treated WT mice. To avoid the intestinal barrier damage caused by colitis, we assessed the intestinal barrier integrity in untreated mice or mice treated with DSS for 2.5 days, in which no colitis symptom difference between WT and *Btbd8* KO mice, including colonic length and histopathology, was observed (**Supplementary Figures S3A–D**). Serum FITC-dextran in WT and *Btbd8* KO mice without DSS treatment is about the same (**Figure 2A**). However, after 2.5-day DSS treatment, serum FITC-dextran in WT mice increases by about 2.5-fold, while it is maintained at a low level in *Btbd8* KO mice (**Figure 2A**). These data indicate that DSS treatment for 2.5 days impairs the barrier integrity in WT mice, allowing more FITC-dextran to enter the serum. However, in *Btbd8* KO mice, the barrier function is strengthened and better maintained. Hence, no significant increase in serum FITC-dextran was detected in *Btbd8* KO mice after 2.5 days of DSS treatment.

### Elevated expression of tight junction proteins in *Btbd8* KO mice

The intestinal barrier is composed of a monolayer of IECs and the mucus layer. Tight junctions (TJs) at the apical region of IECs play a crucial role in maintaining intestinal paracellular permeability and barrier integrity (28–31). We then asked whether TJs in *Btbd8* KO IECs are strengthened to improve the intestinal barrier function. qRT-PCR analysis of isolated IECs revealed increased expression of *ZO-1* and *Occludin* mRNA in *Btbd8* KO IECs (**Figures 2B, C**). However, the mRNA levels of other TJ proteins, including *Claudin-1*, *Claudin-7*, and *E-cadherin*, are not significantly affected by *Btbd8* KO (**Supplementary Figures S3E–G**). Western blot of isolated IECs and immunostaining of colon tissue sections further confirmed that *Btbd8* KO elevates the expression of ZO-1 and Occludin proteins in IECs (**Figures 2D–I**, **Supplementary Figure S3H**). These findings suggest that *Btbd8* deficiency may strengthen TJs and intestinal barrier by increasing the expression of TJ proteins ZO-1 and Occludin.

### *Btbd8* deficiency promotes the activity of ISCs

**Figure 3:**
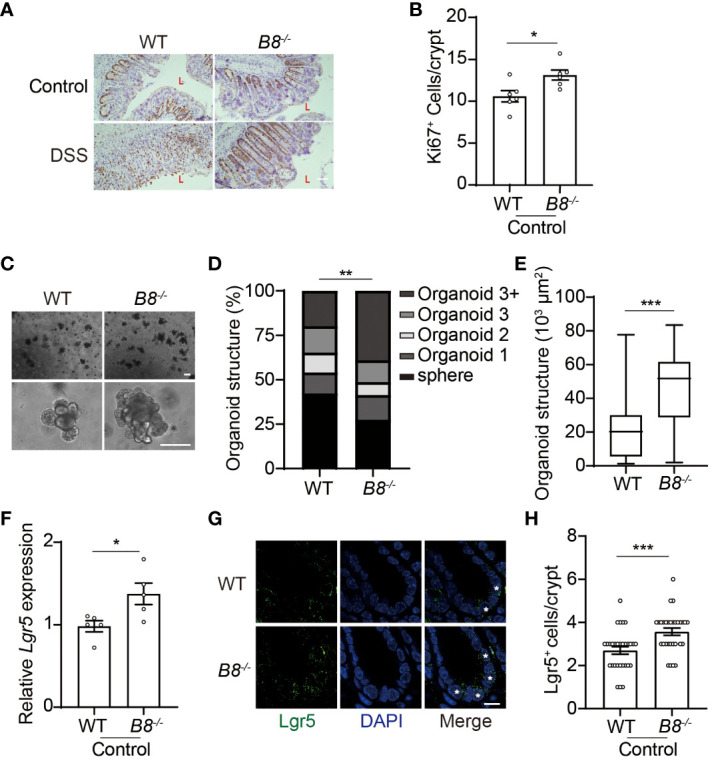
*Btbd8* KO enhances ISC activity. **(A)** Immunohistochemical staining of Ki67 on colonic sections from both WT and *Btbd8* KO mice untreated or treated with DSS for 7 days. The lumen of intestine is indicated by L. Scare bar: 50 μm. **(B)** The number of Ki67^+^ cells per crypt was counted (n=5). **(C)** Representative images of primary intestinal organoids derived from WT and *Btbd8* KO mice after 5 days of culture. Scare bars: 200 μm. **(D)** Organoid structural complexity was assessed by the number of crypt domain per organoid (n>160). **(E)** The size of primary organoid derived from WT and *Btbd8* KO mice (n=73). **(F)** The expression of *Lgr5* in isolated WT and *Btbd8* KO IECs was analyzed by qRT-PCR (n=5). **(G)** Colonic sections from WT and *Btbd8* KO mice were stained with Lgr5. Lgr5^+^ cells are marked by asterisks. Scare bar: 10 μm. **(H)** The number of Lgr5^+^ cells per crypt was quantified from **(G)** (n>30). Data were presented as means ± SEM. *, p < 0.05; **, p < 0.01; ***, p < 0.001.

The intestinal epithelial layer is highly dynamic, with IECs undergoing rapid renewal and replacement approximately every couple of days. The self-renewal and differentiation of Lgr5^+^ ISCs located at the bottom of the intestinal crypts, maintain the homeostasis of the intestinal epithelium (32, 33). Thus, it is possible that enhanced epithelial regeneration contributes to the strengthened intestinal barrier in *Btbd8* KO mice upon DSS treatment. Immunohistochemistry staining of Ki67 reveals more proliferating cells in the colon of *Btbd8* KO mice (**Figures 3A, B**), suggesting a potential role of *Btbd8* in regulating intestinal regeneration. To assess the self-renewal capacity of ISCs, crypts isolated from the intestine were cultured to form intestinal organoids *in vitro*. It is notable that *Btbd8* KO intestinal organoids have more crypt domains per organoid and are of bigger size (**Figures 3C–E**), indicating enhanced self-renewal capacity of *Btbd8* KO ISCs. These observations were replicated in passage 2 organoids (**Supplementary Figure S4**), confirming that *Btbd8* KO promotes the activity of ISCs. Consistently, more *Lgr5* RNA is expressed in *Btbd8* KO IECs (**Figure 3F**). Immunofluorescent staining further revealed that the number of Lgr5^+^ ISCs per crypt is increased in *Btbd8* KO mice (**Figures 3G, H**, **Supplementary Figure S3I**). These data suggest that strengthened intestinal barrier in *Btbd8* KO mice may be partly due to enhanced epithelial regeneration.

### *Btbd8* deficiency elevates TJ protein expression through reducing endocytosis

**Figure 4:**
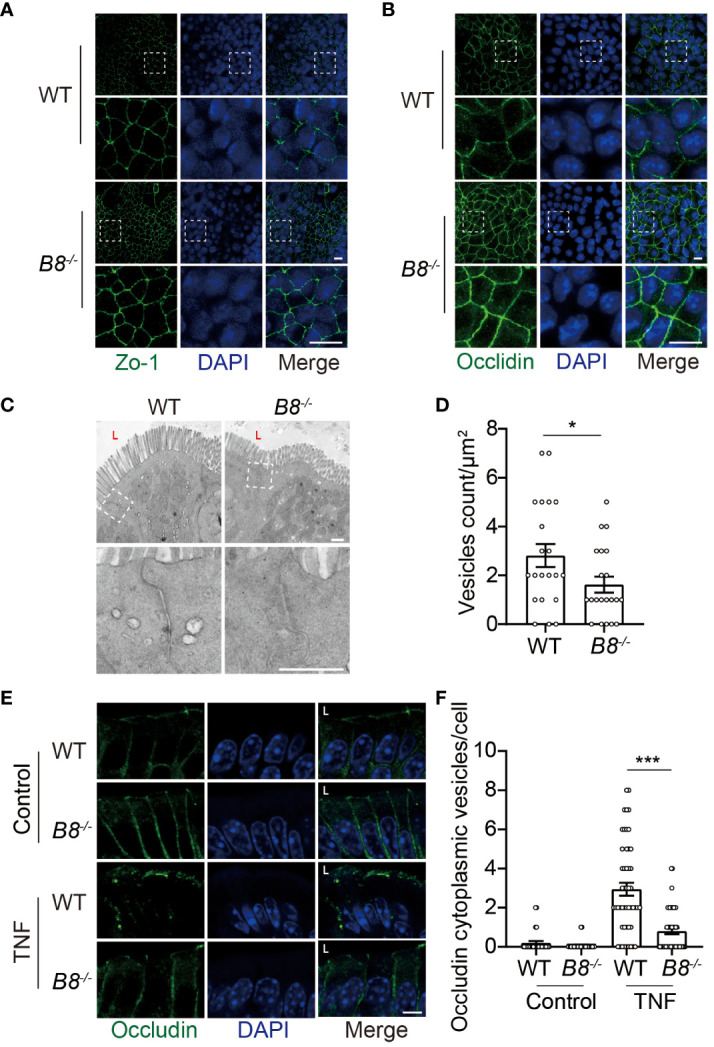
Reduced Occludin internalization, but not enhanced ISC activity, contributes to elevated expression of Occludin in *Btbd8* KO mice. **(A, B)** Immunofluorescence staining of ZO-1 **(A)** and Occludin **(B)** in epithelial monolayer derived from WT and *Btbd8* KO intestinal organoid. Magnified images of boxed regions are shown below the corresponding original images. Scare bar: 10 μm. **(C)** Representative TEM images of TJ regions in colonic enterocytes (n=2). The lumen of intestine is indicated by L. Lower panel: magnified images of boxed regions. Scare bar: 500 nm. **(D)** The number of endocytic vesicles per μm^2^ in TJ regions was quantified from the TEM images in **(C)** (n=21). **(E)** Representative images of Occludin immunofluorescence staining in colonic crypt epithelium of control and TNF treated WT and *Btbd8* KO mice (n=2). The lumen of intestine is indicated by L. Scare bar: 5 μm. **(F)** The count of Occludin containing vesicles in colonic crypt epithelium of control (n=20) and TNF treated (n=50) WT and *Btbd8* KO mice. Data were presented as means ± SEM. *, p < 0.05; ***, p < 0.001.

The enhanced activity of ISCs in *Btbd8* KO mice might account for elevated expression of TJ proteins, because freshly differentiated IECs might express more TJ proteins than worn out IECs. To determine whether the elevated level of ZO-1 and Occludin in *Btbd8* KO IECs is caused by increased ISC activity, epithelial monolayers were derived from WT and *Btbd8* KO organoids, thus avoiding the worn-out effect on IECs. The expression of ZO-1 and Occludin proteins are also increased in *Btbd8* KO epithelial monolayers (**Figures 4A, B**), suggesting that enhanced ISC activity does not account for the elevated expression of ZO-1 and Occludin in *Btbd8* KO IECs.

It has been shown that Btbd8 interacts with AP2, the most abundant adaptor coordinating coat recruitment and cargo selection into endocytic pits, and regulates the endocytosis in synapses (23). Moreover, endocytosis modulates the expression of Occludin in epithelial cells (34, 35). Thus, we speculated that Btbd8 might promote the endocytosis of Occludin to reduce the expression of Occludin in TJs. To assess endocytic activity, we performed transmission electron microscope (TEM) and found that the number of vesicles in the apical region of *Btbd8* KO IECs is reduced, compared with that in WT IECs (**Figures 4C, D**). Next, immunofluorescence assay was applied to examine whether *Btbd8* KO affects the endocytosis of Occludin in IECs. However, no Occludin-containing cytoplasmic vesicles were detected in either WT or *Btbd8* KO IECs (**Figure 4E**). We then treated mice with TNF to induce the endocytosis of Occludin (36). After TNF administration, more Occludin-containing cytoplasmic vesicles are induced in WT IECs than in *Btbd8* KO IECs (**Figures 4E, F**). These data suggest that Btbd8 might promote the endocytosis of Occludin, thus reducing the expression of Occludin in TJs.

### *Btbd8* deficiency increases goblet cell number and *Muc2* expression

**Figure 5:**
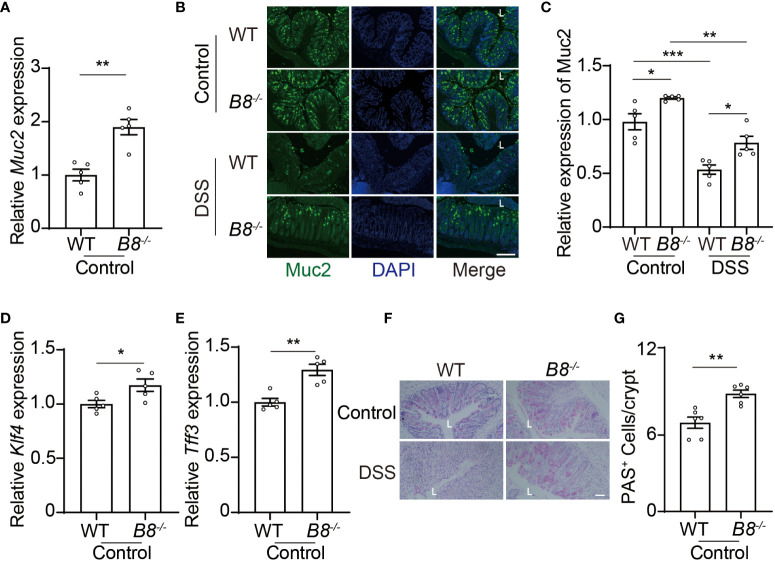
*Btbd8* deficiency increases the number of goblet cells and Muc2 expression. **(A)** The expression of *Muc2* in isolated WT and *Btbd8* KO IECs was analyzed by qRT-PCR (n=5). **(B)** Representative images of Muc2 immunofluorescence staining in colonic sections from control or DSS-treated WT and *Btbd8* KO mice. The lumen of intestine is indicated by L. Scare bar: 200 μm. **(C)** Quantification of Muc2 immunofluorescence (n=5). **(D, E)** The expression of goblet cell markers, *Klf4* **(D)** and *Tff3* **(E)**, in isolated WT and *Btbd8* KO IECs, were assessed by qRT-PCR (n=5). **(F)** Colonic sections from WT and *Btbd8* KO mice were stained with PAS. The lumen of intestine is indicated by L. Scare bar: 50 μm. **(G)** The number of PAS^+^ goblet cells per crypt was counted (n=6). Data were presented as means ± SEM. *, p < 0.05; **, p < 0.01; ***, p < 0.001.

In addition to the physical barrier formed by IECs, the mucus, covering IECs, serves as the foremost defense line to restrict the entry of gut contents into the body (37). Next, we tested the possibility that *Btbd8* KO might strengthen the intestinal barrier by enhancing the function of the mucus. Indeed, the expression of *Muc2* RNA and protein, a major gel-forming mucin in the colon mucus, is increased in *Btbd8* KO IECs (**Figures 5A–C**). Muc2 is predominantly secreted by goblet cells. Thus, we examined the expression of genes critical for goblet cell differentiation, *Klf4* and *Tff3*, and found that *Btbd8* KO elevates the expression of *Klf4* and *Tff3* RNA in IECs (**Figures 5D, E**). Furthermore, the number of acidic mucin-filled goblet cells, as indicated by Periodic Acid-Schiff (PAS) staining, is increased in *Btbd8* KO mice (**Figures 5F, G**). These data suggest that *Btbd8* deficiency might promote the development of goblet cells and enhance the function of the mucus to improve the intestinal barrier.

### *Btbd8* deficiency reduces pro-inflammation cytokine expression in macrophages

**Figure 6:**
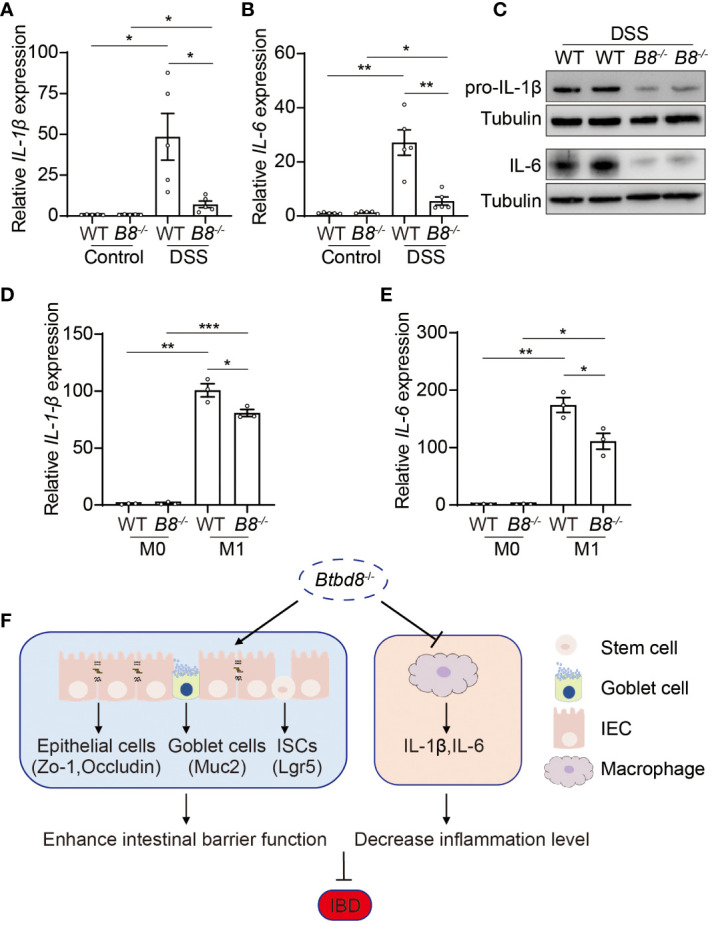
Reduced expression of pro-inflammatory cytokines, IL-1β and IL-6, by *Btbd8* KO macrophages. **(A, B)** The expression of *IL-1β* **(A)** and *IL-6* **(B)** in colon tissues of control or DSS-treated WT and *Btbd8* KO mice (n=5). **(C)** Expression of IL-pro-1β and IL-6 proteins in colon tissues from DSS-treated WT and *Btbd8* KO mice were detected by Western blot. **(D, E)** The expression of *IL-1β* **(D)** and *IL-6* **(E)** in WT and *Btbd8* KO BMDMs stimulated with PBS or LPS/IFN-γ was analyzed by qRT-PCR (n=3). **(F)** A working model for Btbd8 to regulate IBD pathogenesis. *Btbd8* KO promotes the expression of TJ proteins ZO-1 and Occludin, enhances ISC activity, and increases the number of goblet cells and Muc2 secretion, thus improving the intestinal barrier integrity. In addition, loss of *Btbd8* reduces the expression of *IL-1β* and *IL-6* by macrophages, hence mitigating inflammation. Collectively, *Btbd8* deficiency confers decreased susceptibility to colitis. Data were presented as means ± SEM. *, p < 0.05; **, p < 0.01; ***, p < 0.001.

The development of IBD is closely associated with the progression of inflammation (2, 30, 38). Macrophages play a pivotal role in innate immune defense by producing cytokines, such as IL-1β and IL-6, which initiate and exacerbate inflammation (39, 40). Moreover, a low frequency variant of *BTBD8* has been shown to associate with reduced monocyte counts, which give rise to mature macrophages (41). We then evaluated whether Btbd8 affects the function of macrophages, in addition to intestinal barrier integrity. qRT-PCR and Western blot revealed a significant reduction in both RNA and protein levels of IL-1β and IL-6 in the colonic tissues of DSS treated *Btbd8* KO mice, compared with DSS treated WT mice (**Figures 6A–C**). The reduced expression of IL-1β and IL-6 might be due to the defect of *Btbd8* KO macrophages or milder inflammation in DSS treated *Btbd8* KO mice. To validate whether *Btbd8* KO leads to intrinsic defect of macrophages, bone marrow derived macrophages (BMDMs) isolated from WT and *Btbd8* KO mice were stimulated with lipopolysaccharide (LPS) and IFN-γ *in vitro*. Consistent with the *in vivo* data, the expression of *IL-1β* and *IL-6* after LPS and IFN-γ treatment was lower in *Btbd8* KO BMDMs, than in WT BMDMs (**Figures 6D, E**), indicating intrinsic defect of *Btbd8* KO macrophages in response to external stimuli. Taken together, our data demonstrate that *Btbd8* KO reduces the expression of IL-1β and IL-6 in macrophages in response to *in vivo* DSS treatment or *in vitro* LPS/IFN-γ stimulation.

## Discussion

Even though *BTBD8* has been identified as an IBD susceptibility gene, its role in normal development and IBD pathogenesis remains unclear. In this study, we knocked out *Btbd8* in mice, and no obvious phenotype was noted in *Btbd8* KO mice. Nevertheless, *Btbd8* KO mice are partially resistant to DSS-induced IBD, confirming the function of Btbd8 in IBD pathogenesis. In addition, the expression of *BTBD8* RNA is reduced in inflamed tissues of UC patients, compared with those in non-inflamed tissues of UC patients and in healthy individuals (**Supplementary Figure S2B**). DSS treatment also leads to decreased level of *Btbd8* RNA in mice (**Supplementary Figure S2C**). These data suggest that under disease conditions, *Btbd8* might be downregulated to prevent the progression of IBD.

Btbd8 regulates IBD pathogenesis through multiple mechanisms. First, *Btbd8* depletion enhances the intestinal barrier function, including the epithelium and the mucus. TJ proteins ZO-1 and Occludin are upregulated in *Btbd8* KO IECs, indicating stronger TJs. Therefore, intestinal barrier integrity is better maintained, particularly upon DSS treatment. In addition, elevated ISC activity in *Btbd8* KO mice allowing more efficient epithelial regeneration, might contribute to the maintenance of intestinal barrier integrity. *Btbd8* deficiency also leads to increased number of goblet cells, which secret Muc2 to form the mucus layer. Thus, the barrier function of the mucus is strengthened in *Btbd8* KO mice. Secondly, *Btbd8* deficiency may suppress inflammation through reducing pro-inflammatory cytokine expression in macrophages, such as IL-1β and IL-6, thus ameliorating the DSS-induced IBD symptom (**Figure 6F**).

However, how Btbd8 regulates the expression of ZO-1, Occludin, Muc2, IL-1β and IL-6, remains elusive. The expression of these genes is upregulated at both RNA and protein levels in *Btbd8* KO mice, suggesting that Btbd8 might act as a transcription factor or co-factor to regulate the transcription of these genes. Consistent with this note, Btbd8 contains double BTB/POZ domains, which are often found in developmentally regulated transcription factors (42). In addition, Btbd8 may promote endocytosis to suppress the expression of Occludin in TJs. These two distinct mechanisms require Btbd8 to function in different subcellular compartments, the nucleus and endocytic vesicles. Unfortunately, we did not have a good antibody for detecting Btbd8 protein. Therefore, we were unable to experimentally demonstrate the subcellular distribution of Btbd8 in IECs, goblet cells, and macrophages. Nevertheless, protein interactome studies have revealed that Btbd8 interact with not only nuclear proteins, but also cytoplasmic proteins, particularly AP2 the main adaptor protein responsible for clathrin-mediated endocytosis (23, 43, 44), implying that Btbd8 might function as a transcription factor, as well as an endocytosis regulator. Moreover, NF-κB activation rapidly induces the expression of pro-IL-1β, which is subsequently converted into its active form, IL-1β, by caspase-1 within the inflammasome (45, 46). These processes play a pivotal role in DSS-induced colitis. Given that *Btbd8* KO leads to enhanced expression of pro-IL-1β in the colon, it is worth to investigate whether Btbd8 is involved in activating NF-κB, and whether Btbd8 regulates the processing of pro-IL-1β by caspase-1 and inflammasomes. Additionally, a low-frequency variant of *BTBD8* has been associated with reduced monocyte counts, potentially related to less mature macrophages (41). It implies that the KO of *Btbd8* may reduce the number of colonic macrophages, which needs to be validated experimentally.

In summary, our studies revealed the role of *Btbd8* in IBD pathogenesis. The reduced susceptibility to DSS-induced colitis in *Btbd8* KO mice may attribute to enhanced intestinal barrier function and suppressed inflammation. Thus, Btbd8 might be a potential therapeutic target for IBD treatment.

## Data availability statement

The raw data supporting the conclusions of this article will be made available by the authors, without undue reservation.

## Ethics statement

The animal study was approved by Nankai Animal Care and Use Committee (Approval number: 2021-SYDWLL-1-0001). The study was conducted in accordance with the local legislation and institutional requirements.

## Author contributions

XY: Data curation, Investigation, Methodology, Software, Supervision, Validation, Visualization, Writing – original draft, Writing – review & editing. ZH: Investigation, Methodology, Writing – review & editing. QD: Investigation, Methodology, Writing – review & editing. SN: Investigation, Methodology, Writing – review & editing. XWD: Investigation, Writing – review & editing. JY: Investigation, Writing – review & editing. NZ: Methodology, Writing – review & editing. XLD: Writing – review & editing, Investigation. LC: Conceptualization, Data curation, Funding acquisition, Investigation, Supervision, Validation, Writing – original draft, Writing – review & editing.

## References

[1] Andersson-Rolf A Zilbauer M Koo BK Clevers H. Stem cells in repair of gastrointestinal epithelia. Physiol (Bethesda). (2017) 32:278–89. doi: 10.1152/physiol.00005.2017 PMC5545610 28615312

[2] Rogler G Andus T. Cytokines in inflammatory bowel disease. World J Surg. (1998) 22:382–9. doi: 10.1007/s002689900401 9523521

[3] Hold GL Smith M Grange C Watt ER El-Omar EM Mukhopadhya I. Role of the gut microbiota in inflammatory bowel disease pathogenesis: what have we learnt in the past 10 years? World J Gastroenterol. (2014) 20:1192–210. doi: 10.3748/wjg.v20.i5.1192 PMC3921503 24574795

[4] Ramos GP Papadakis KA. Mechanisms of disease: inflammatory bowel diseases. Mayo Clin Proc. (2019) 94:155–65. doi: 10.1016/j.mayocp.2018.09.013 PMC6386158 30611442

[5] Larabi A Barnich N Nguyen HTT. New insights into the interplay between autophagy, gut microbiota and inflammatory responses in ibd. Autophagy. (2020) 16:38–51. doi: 10.1080/15548627.2019.1635384 31286804 PMC6984609

[6] Gao X Cao Q Cheng Y Zhao D Wang Z Yang H. Chronic stress promotes colitis by disturbing the gut microbiota and triggering immune system response. Proc Natl Acad Sci USA. (2018) 115:E2960–E9. doi: 10.1073/pnas.1720696115 PMC5879702 29531080

[7] Liu JZ van Sommeren S Huang H Ng SC Alberts R Takahashi A. Association analyses identify 38 susceptibility loci for inflammatory bowel disease and highlight shared genetic risk across populations. Nat Genet. (2015) 47:979–86. doi: 10.1038/ng.3359 PMC4881818 26192919

[8] Jostins L Ripke S Weersma RK Duerr RH McGovern DP Hui KY. Host-microbe interactions have shaped the genetic architecture of inflammatory bowel disease. Nature. (2012) 491:119–24. doi: 10.1038/nature11582 PMC3491803 23128233

[9] de Lange KM Moutsianas L Lee JC Lamb CA Luo Y Kennedy NA. Genome-wide association study implicates immune activation of multiple integrin genes in inflammatory bowel disease. Nat Genet. (2017) 49:256–61. doi: 10.1038/ng.3760 PMC5289481 28067908

[10] Huang H Fang M Jostins L Umicevic Mirkov M Boucher G Anderson CA. Fine-mapping inflammatory bowel disease loci to single-variant resolution. Nature. (2017) 547:173–8. doi: 10.1038/nature22969 PMC5511510 28658209

[11] Mohanan V Nakata T Desch AN Levesque C Boroughs A Guzman G. C1orf106 is a colitis risk gene that regulates stability of epithelial adherens junctions. Science. (2018) 359:1161–6. doi: 10.1126/science.aan0814 PMC6008784 29420262

[12] Fujimoto K Kinoshita M Tanaka H Okuzaki D Shimada Y Kayama H. Regulation of intestinal homeostasis by the ulcerative colitis-associated gene rnf186. Mucosal Immunol. (2017) 10:446–59. doi: 10.1038/mi.2016.58 27381925

[13] Darsigny M Babeu JP Dupuis AA Furth EE Seidman EG Levy E. Loss of hepatocyte-nuclear-factor-4alpha affects colonic ion transport and causes chronic inflammation resembling inflammatory bowel disease in mice. PloS One. (2009) 4:e7609. doi: 10.1371/journal.pone.0007609 19898610 PMC2764139

[14] Cattin AL Le Beyec J Barreau F Saint-Just S Houllier A Gonzalez FJ. Hepatocyte nuclear factor 4alpha, a key factor for homeostasis, cell architecture, and barrier function of the adult intestinal epithelium. Mol Cell Biol. (2009) 29:6294–308. doi: 10.1128/MCB.00939-09 PMC2786690 19805521

[15] Pabst O Zweigerdt R Arnold HH. Targeted disruption of the homeobox transcription factor nkx2-3 in mice results in postnatal lethality and abnormal development of small intestine and spleen. Development. (1999) 126:2215–25. doi: 10.1242/dev.126.10.2215 10207146

[16] Pickert G Neufert C Leppkes M Zheng Y Wittkopf N Warntjen M. Stat3 links il-22 signaling in intestinal epithelial cells to mucosal wound healing. J Exp Med. (2009) 206:1465–72. doi: 10.1084/jem.20082683 PMC2715097 19564350

[17] Lamas B Richard ML Leducq V Pham HP Michel ML Da Costa G. Card9 impacts colitis by altering gut microbiota metabolism of tryptophan into aryl hydrocarbon receptor ligands. Nat Med. (2016) 22:598–605. doi: 10.1038/nm.4102 27158904 PMC5087285

[18] Cooney R Baker J Brain O Danis B Pichulik T Allan P. Nod2 stimulation induces autophagy in dendritic cells influencing bacterial handling and antigen presentation. Nat Med. (2010) 16:90–7. doi: 10.1038/nm.2069 19966812

[19] Ogura Y Bonen DK Inohara N Nicolae DL Chen FF Ramos R. A frameshift mutation in nod2 associated with susceptibility to crohn’s disease. Nature. (2001) 411:603–6. doi: 10.1038/35079114 11385577

[20] Kuhn R Lohler J Rennick D Rajewsky K Muller W. Interleukin-10-deficient mice develop chronic enterocolitis. Cell. (1993) 75:263–74. doi: 10.1016/0092-8674(93)80068-P 8402911

[21] Duerr RH Taylor KD Brant SR Rioux JD Silverberg MS Daly MJ. A genome-wide association study identifies il23r as an inflammatory bowel disease gene. Science. (2006) 314:1461–3. doi: 10.1126/science.1135245 PMC4410764 17068223

[22] Smillie CS Biton M Ordovas-Montanes J Sullivan KM Burgin G Graham DB. Intra- and inter-cellular rewiring of the human colon during ulcerative colitis. Cell. (2019) 178:714–30 e22. doi: 10.1016/j.cell.2019.06.029 31348891 PMC6662628

[23] Piccini A Castroflorio E Valente P Guarnieri FC Aprile D Michetti C. Apache is an ap2-interacting protein involved in synaptic vesicle trafficking and neuronal development. Cell Rep. (2017) 21:3596–611. doi: 10.1016/j.celrep.2017.11.073 29262337

[24] Wirtz S Popp V Kindermann M Gerlach K Weigmann B Fichtner-. Chemically induced mouse models of acute and chronic intestinal inflammation. Nat Protoc. (2017) 12:1295–309. doi: 10.1038/nprot.2017.044 28569761

[25] Arranz A Doxaki C Vergadi E Martinez de la Torre Y Vaporidi K Lagoudaki ED. Akt1 and akt2 protein kinases differentially contribute to macrophage polarization. Proc Natl Acad Sci USA. (2012) 109:9517–22. doi: 10.1073/pnas.1119038109 PMC3386059 22647600

[26] Grabinger T Luks L Kostadinova F Zimberlin C Medema JP Leist M. Ex vivo culture of intestinal crypt organoids as a model system for assessing cell death induction in intestinal epithelial cells and enteropathy. Cell Death Dis. (2014) 5:e1228. doi: 10.1038/cddis.2014.183 24832600 PMC4047863

[27] Liao L Dang W Lin T Yu J Liu T Li W. A potent pgk1 antagonist reveals pgk1 regulates the production of il-1beta and il-6. Acta Pharm Sin B. (2022) 12:4180–92. doi: 10.1016/j.apsb.2022.05.012 PMC9643279 36386479

[28] Peterson LW Artis D. Intestinal epithelial cells: regulators of barrier function and immune homeostasis. Nat Rev Immunol. (2014) 14:141–53. doi: 10.1038/nri3608 24566914

[29] Gierynska M Szulc-Dabrowska L Struzik J Mielcarska MB Gregorczyk-Zboroch KP. Integrity of the intestinal barrier: the involvement of epithelial cells and microbiota-a mutual relationship. Anim (Basel). (2022) 12:145. doi: 10.3390/ani12020145 PMC8772550 35049768

[30] Chen Y Cui W Li X Yang H. Interaction between commensal bacteria, immune response and the intestinal barrier in inflammatory bowel disease. Front Immunol. (2021) 12:761981. doi: 10.3389/fimmu.2021.761981 34858414 PMC8632219

[31] Chelakkot C Ghim J Ryu SH. Mechanisms regulating intestinal barrier integrity and its pathological implications. Exp Mol Med. (2018) 50:1–9. doi: 10.1038/s12276-018-0126-x PMC6095905 30115904

[32] Clevers H. The intestinal crypt, a prototype stem cell compartment. Cell. (2013) 154:274–84. doi: 10.1016/j.cell.2013.07.004 23870119

[33] van der Flier LG Clevers H. Stem cells, self-renewal, and differentiation in the intestinal epithelium. Annu Rev Physiol. (2009) 71:241–60. doi: 10.1146/annurev.physiol.010908.163145 18808327

[34] Ivanov AI Nusrat A Parkos CA. Endocytosis of epithelial apical junctional proteins by a clathrin-mediated pathway into a unique storage compartment. Mol Biol Cell. (2004) 15:176–88. doi: 10.1091/mbc.e03-05-0319 PMC307538 14528017

[35] Fletcher SJ Poulter NS Haining EJ Rappoport JZ. Clathrin-mediated endocytosis regulates occludin, and not focal adhesion, distribution during epithelial wound healing. Biol Cell. (2012) 104:238–56. doi: 10.1111/boc.201100004 22187938

[36] Marchiando AM Shen L Graham WV Weber CR Schwarz BT Austin JR 2nd. Caveolin-1-dependent occludin endocytosis is required for tnf-induced tight junction regulation in vivo. J Cell Biol. (2010) 189:111–26. doi: 10.1083/jcb.200902153 PMC2854371 20351069

[37] Johansson MEV Phillipson M Petersson J Velcich A Holm L Hansson GC. The inner of the two muc2 mucin-dependent mucus layers in colon is devoid of bacteria. Proc Natl Acad Sci USA. (2008) 105:15064–9. doi: 10.1073/pnas.0803124105 PMC2567493 18806221

[38] Tatiya-Aphiradee N Chatuphonprasert W Jarukamjorn K. Immune response and inflammatory pathway of ulcerative colitis. J Basic Clin Physiol Pharmacol. (2018) 30:1–10. doi: 10.1515/jbcpp-2018-0036 30063466

[39] Na YR Stakenborg M Seok SH Matteoli G. Macrophages in intestinal inflammation and resolution: A potential therapeutic target in ibd. Nat Rev Gastroenterol Hepatol. (2019) 16:531–43. doi: 10.1038/s41575-019-0172-4 31312042

[40] Pan X Zhu Q Pan LL Sun J. Macrophage immunometabolism in inflammatory bowel diseases: from pathogenesis to therapy. Pharmacol Ther. (2022) 238:108176. doi: 10.1016/j.pharmthera.2022.108176 35346728

[41] Group CCHW. Meta-analysis of rare and common exome chip variants identifies S1pr4 and other loci influencing blood cell traits. Nat Genet. (2016) 48:867–76. doi: 10.1038/ng.3607 PMC5145000 27399967

[42] Xu J He T Wang L Wu Q Zhao E Wu M. Molecular cloning and characterization of a novel human btbd8 gene containing double btb/poz domains. Int J Mol Med. (2004) 13:193–7. doi: 10.3892/ijmm 14654994

[43] Fasci D van Ingen H Scheltema RA Heck AJR. Histone interaction landscapes visualized by crosslinking mass spectrometry in intact cell nuclei. Mol Cell Proteomics: MCP. (2018) 17:2018–33. doi: 10.1074/mcp.RA118.000924 PMC6166682 30021884

[44] Hein MY Hubner NC Poser I Cox J Nagaraj N Toyoda Y. A human interactome in three quantitative dimensions organized by stoichiometries and abundances. Cell. (2015) 163:712–23. doi: 10.1016/j.cell.2015.09.053 26496610

[45] Siegmund B Lehr HA Fantuzzi G Dinarello CA. Il-1 beta -converting enzyme (Caspase-1) in intestinal inflammation. Proc Natl Acad Sci U.S.A. (2001) 98:13249–54. doi: 10.1073/pnas.231473998 PMC60856 11606779

[46] Masters SL Simon A Aksentijevich I Kastner DL. Horror autoinflammaticus: the molecular pathophysiology of autoinflammatory disease (*). Annu Rev Immunol. (2009) 27:621–68. doi: 10.1146/annurev.immunol.25.022106.141627 PMC2996236 19302049

