# Supplementary material for: Btbd8 deficiency reduces susceptibility to colitis by enhancing intestinal barrier function and suppressing inflammation
Source: Front Immunol. 2024 Mar 15;15:1382661. doi: 10.3389/fimmu.2024.1382661 (PMC10978791; doi:10.3389/fimmu.2024.1382661)
Supplement: Supplementary file 1 [file DataSheet_1.docx]

Supplementary Material

# Supplementary Figures and Tables

## Supplementary Figures

**
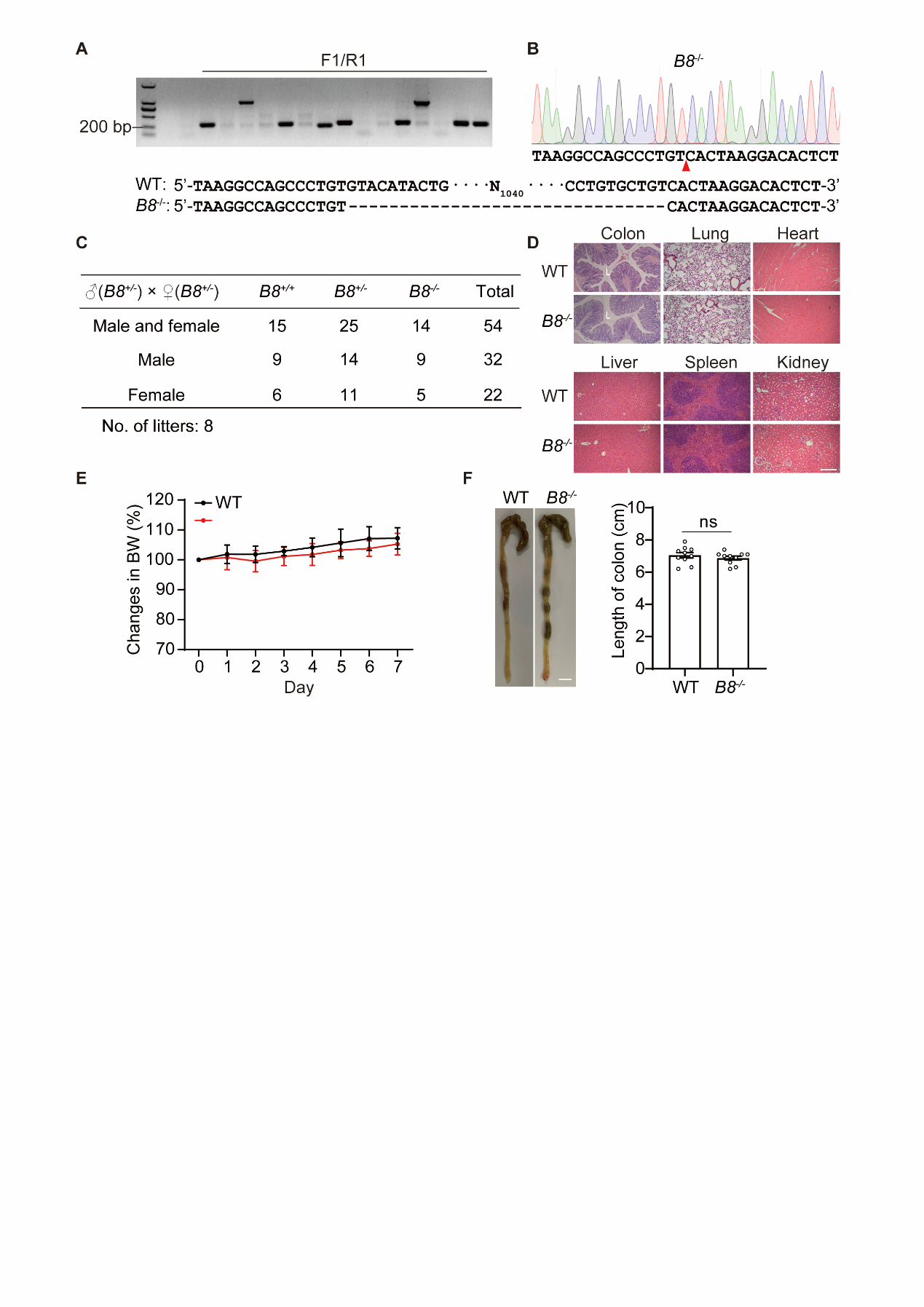
**

**Supplementary Figure 1. *Btbd8* KO mice are healthy and fertile. (A)** Genotyping of the founder mice, using F1/R1 primers shown in Figure 1 (A). **(B)** Sequencing chromatograph of one founder mouse validates the homozygous knockout of *Btbd8*. The red triangle indicates the deletion site. **(C)** Genotypes of the offspring from the mating between *Btbd8*^+/-^ mice. **(D)** 8-week-old WT and *Btbd8* KO mice were sacrificed, and their major organs, including colon, lung, heart, liver, spleen, kidney, were separated for histological examination. The lumen of intestine is indicated by L. Scare bar: 200 μm. **(E)** Body weight changes in WT and *Btbd8* KO mice without DSS treatment (n=7). **(F)** Representative images of colon (left panel) and the length of colon (right panel) in WT and *Btbd8* KO mice without DSS treatment (n=10). Scale bar: 0.5 cm. Data were presented as means ± SEM. ns: not significant.


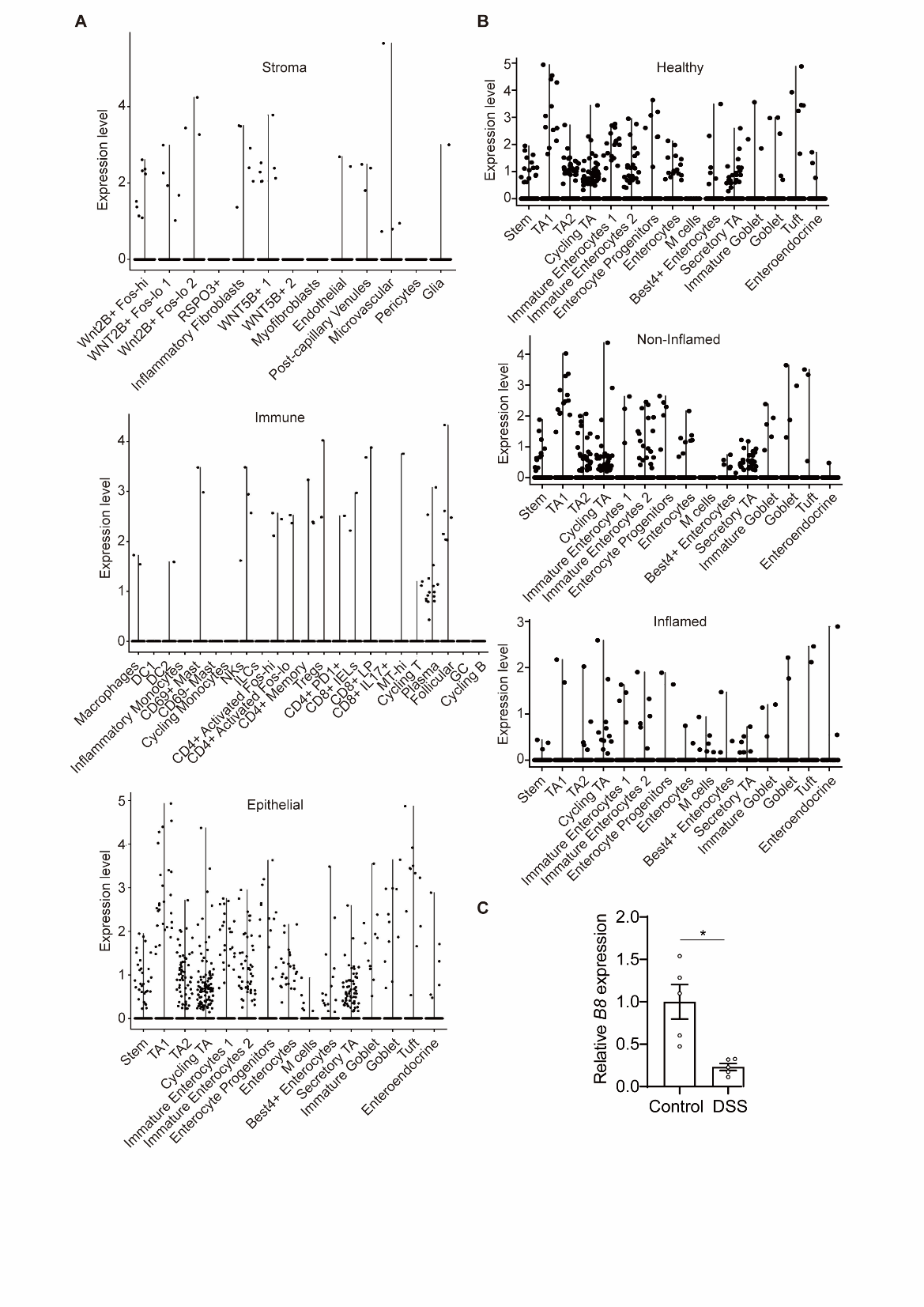


**Supplementary Figure 2. *BTBD8* expression is decreased in the IECs of UC patients.** (**A)** The expression of *BTBD8* in human stroma (top), immune (middle), and epithelial cells (low), extracted from single-cell RNA-seq data (accession number SCP259)(1). (**B)** The expression of *BTBD8* in various epithelial cells from healthy individuals (top), non-inflamed (middle) and inflamed colon tissues (low) of UC patients, extracted from single-cell RNA-seq data (accession number SCP259) (1). (**C)** The expression of *Btbd8* in colons of control or DSS-treated WT mice (n=5). Data were presented as means ± SEM. *, p < 0.05.

**
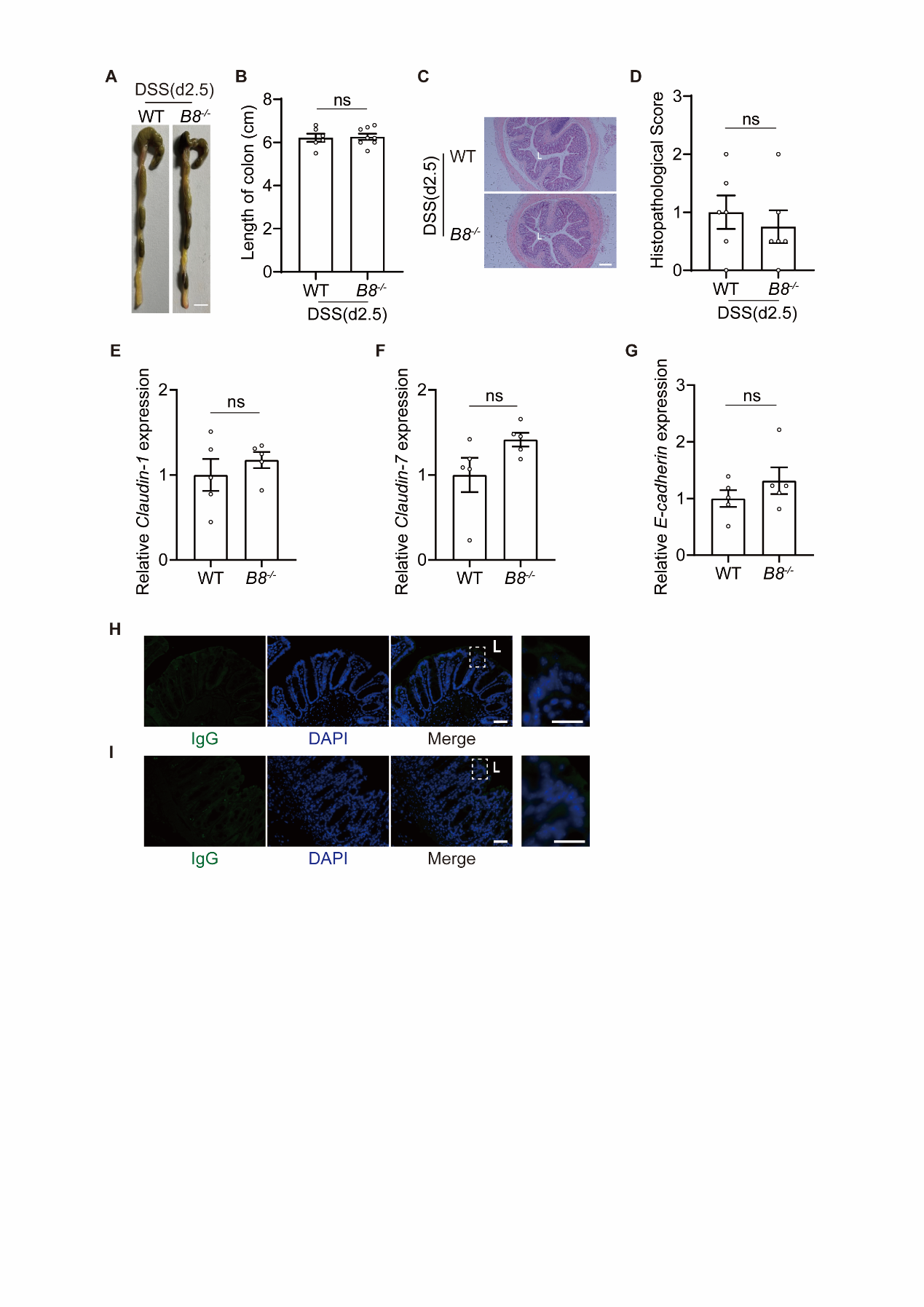
**

**Supplementary Figure 3.** **No morphological and histopathological differences in the colon of WT and *Btbd8* KO mice treated with DSS for 2.5 days. (A)** and **(B)** Representative images of colon (A) and the length of colon (B) in WT (n=6) and *Btbd8* KO (n=8) mice after 2.5-day DSS treatment (n=10). Scale bar: 0.5 cm. **(C)** and **(D)** Representative histopathological images of colon tissue sections (C) and the histopathological score (D) in WT and *Btbd8* KO mice after 2.5-day DSS treatment (n = 6). The lumen of intestine is indicated by L. Scale bar: 200 μm. **(E) to (G)** The expression of *Claduin-1* (E), *Claudin-7* (F) and *E-cadherin* (G) mRNA in isolated WT and *Btbd8* KO IECs was analyzed by qRT-PCR (n=5). (**H**) and (**I**) Rabbit IgG (H) and mouse IgG (I) isotypr control for immunofluorescence staining in colonic sections. The lumen of intestine is indicated by L. Scale bar: 50 μm. Data were presented as means ± SEM. ns: not significant.

**
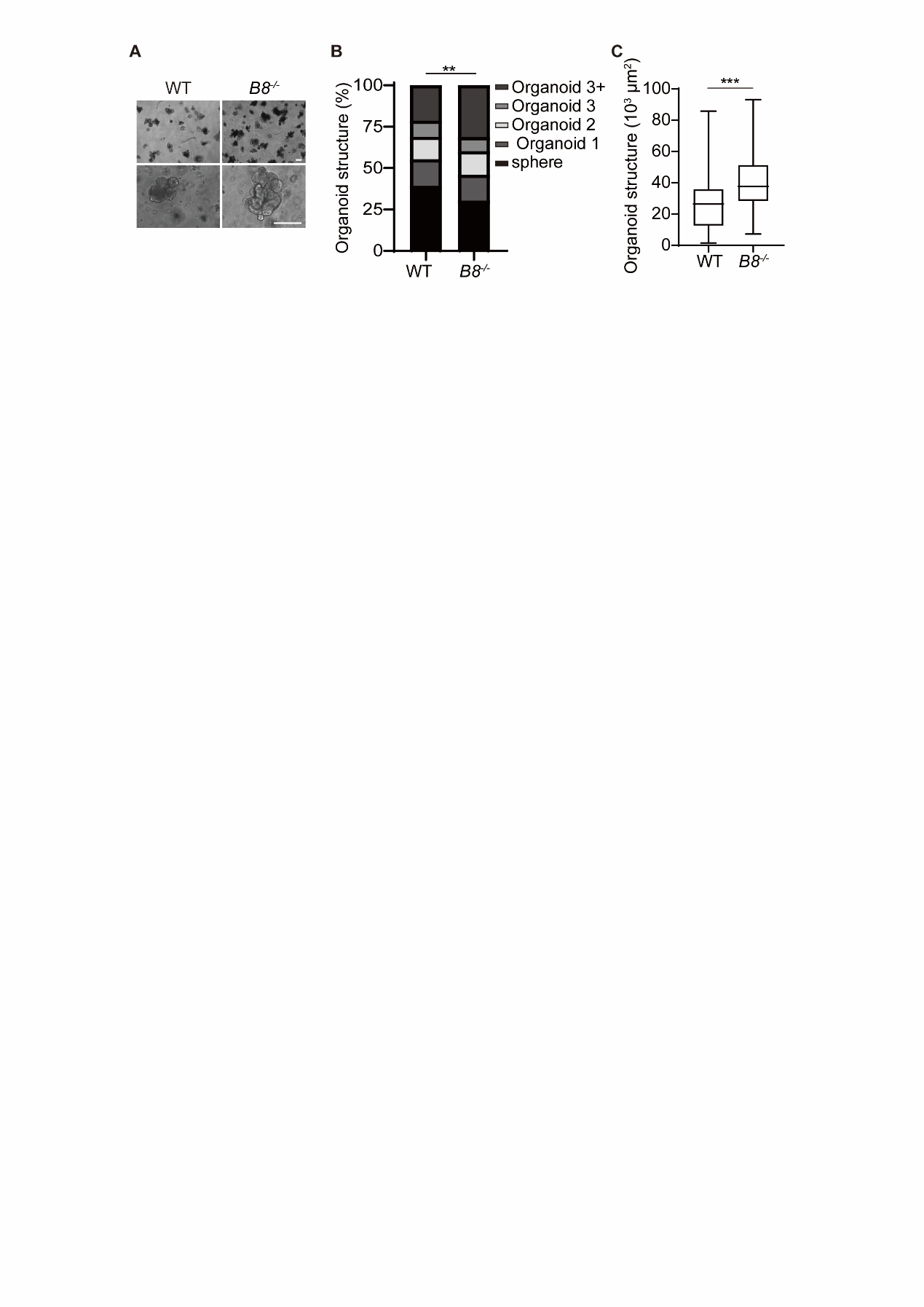
**

**Supplementary Figure 4. *Btbd8* deficiency enhances the growth of second-generation organoids. (A)** Representative images of second-generation intestinal organoids from WT and *Btbd8* KO mice after 5 days culture. Scare bars: 500 μm. **(B)** Organoid structural complexity was assessed by the number of crypt domain per second-generation organoid (n>400). **(C)** The size of second-generation organoid derived from WT and *Btbd8* KO mice (n>55). Data were presented as means ± SEM. **, p < 0.01; ***, p < 0.001.

## Supplementary Table

**Table S1. Primers used in this study**

|  | Gene | Forward | Reverse |
| --- | --- | --- | --- |
| qRT-PCR primers | *Btbd8* | GGTACTCACTGCAAACCTGAA | CCGTGCTGGAATGTGTAGAT |
|  | *ZO-1* | GAGCGGGCTACCTTACTGAAC | GTCATCTCTTTCCGAGGCATTAG |
|  | *Occludin* | ACGGACCCTGACCACTATGA | TCAGCAGCAGCCATGTACTC |
|  | *Muc2* | ATGCCCACCTCCTCAAAGAC | GTAGTTTCCGTTGGAACAGTGAA |
|  | *Klf4* | AGCCACCCACACTTGTGACTAT | AGTGGTAAGGTTTCTCGCCTGT |
|  | *Tff3* | TTGCTGGGTCCTCTGGGATAG | TACACTGCTCCGATGTGACAG |
|  | *Lgr5* | CGAGCCTTACAGAGCCTGATACC | TTGCCGTCGTCTTTATTCCATTGG |
|  | *IL-1β* | GAAATGCCACCTTTTGACAGTG | TGGATGCTCTCATCAGGACAG |
|  | *IL-6* | ACTTCCATCCAGTTGCCTTCTTGG | TTAAGCCTCCGACTTGTGAAGTGG |
|  | *Gapdh* | TGTGTCCGTCGTGGATCTGA | CCTGCTTCACCACCTTCTTGA |
| Genotyping primers | *Btbd8*-F1/R1 | GCTTTGCTCAATTGTTGCTTCT | TTGCCTGGCTTCGTCTCTA |
|  | *Btbd8*-F2/R2 | CATCCACTGTTGTGGGAAGA | CCGTGCTGGAATGTGTAGAT |

**Supplementary References**

1. Smillie CS, Biton M, Ordovas-Montanes J, Sullivan KM, Burgin G, Graham DB, et al. Intra- and Inter-Cellular Rewiring of the Human Colon During Ulcerative Colitis. *Cell* (2019) 178(3):714-30 e22. doi: 10.1016/j.cell.2019.06.029.
